# Authoritative parenting style positively correlates with increased adherence to COVID-19 health and safety guidelines by college students

**DOI:** 10.3389/fpsyg.2023.1085763

**Published:** 2023-03-07

**Authors:** Adrienne J. Spivey, Jessica Stagner Bodily

**Affiliations:** Department of Psychology, Auburn University at Montgomery, Montgomery, AL, United States

**Keywords:** COVID-19, parenting style, choice, behavior, health, compliance

## Abstract

During the COVID-19 pandemic, there has been inconsistency in choice behavior across people with regards to compliance with health and safety guidelines suggested by the CDC. The current study aimed to identify a possible correlation between parenting style experienced during childhood and opinions/actions regarding CDC COVID-19 health guidance. College students were given a self-report survey aimed to measure childhood experience, parent–child relationship, and COVID-19 pandemic behavior. Participants that identified with Authoritarian parenting scored higher on the COVID Behavior measure, indicating a higher degree of compliance compared to participants that identified with Authoritative parenting. Additionally, gender and race category differences on the COVID Behavior measure were observed. Specifically, African American/Black participants scored higher than White or Other race identifying participants on the COVID Behavior measure. Lastly, females identifying as African American/Black or Other race scored higher than males identifying as African American/Black or Other race on the COVID Behavior measure. These findings begin to illuminate some of the variables that might play a role in choice behavior with regard to compliance to health guidelines. Further investigation into these variables could inform us about what plays a role in choice behavior and how better to integrate this knowledge when messaging the public about health guidelines.

## Introduction

In 2019, the COVID-19 virus was identified through an outbreak of respiratory illness first detected in Wuhan, Hubei province, China. This specific strain of coronavirus had not been previously identified in humans and has only spread through contact between a human and an animal carrying the disease (CDC, 2019). Since December 2019, cases have been identified in a growing number of countries, including the United States. Symptoms of COVID-19 include cough, fever, headache, loss of taste or smell, shaking, chills, sore throat, shortness of breath, muscle pain, and even death in severe cases (CDC, 2019). In early 2020, a global COVID-19 pandemic was declared, which resulted in new safety protocols in public spaces and private businesses. These protocols included suggestions for a mandatory distance of at least six feet between individuals, as well as the use of facial masks when indoors and/or in large crowds (Centers for Disease Control and Prevention, 2020; World Health Organization, 2021).

On November 20, 2020, The CDC issued an MMWR (Morbidity and Mortality Weekly Report) Early Release on the MMWR website stating that mask-wearing was a recommended action to reduce the spread of the COVID-19 virus because masks prevent inhalation of respiratory droplets from coughs, sneezes, and speech (Centers for Disease Control and Prevention, 2020; World Health Organization, 2021). Despite this recommendation, some individuals were resistant to adhere. In southwest Iran, a cross-sectional study observing the usage of facial masks during the COVID-19 pandemic was conducted. 10,440 pedestrians were selected from eight urban districts and 92 neighborhoods of the city. Most of the sample consisted of male participants (67.9%) aged from 10–39 years. In the entire sample, only 45.6% of participants wore face masks, with females wearing them more often than males (60.2% vs. 38.7%). In addition, it was found that as age increased, so did face mask use. Participants wore face masks more often in the a.m. (49.4%) than in the p.m. (43.9%). Lastly, 75.6% of the entire sample wore face masks correctly (Rahimi et al., 2021).

To examine proper mask wear, another study was done in Tel-Aviv in which pedestrians were approached and offered a monetary incentive for performing the die-under-the-cup (DUTC) task (Tobol et al., 2020). The DUTC task rewards dishonest behavior because the participant rolls a 6-sided die and reports the number they land on, then receives that amount of money (so, for example, if a six was rolled then six dollars was awarded). This experiment was meant to test whether people who wore masks improperly were more likely to lie than those who wore masks properly or wore no mask at all. The total sample consisted of 325 people –113 correctly wearing masks, 105 improperly wearing masks, and 107 wearing no mask. When participant scores were compared to “the average expected outcome of 3.5 in a fair die roll,” the people wearing masks improperly had a higher mean die outcome than the other groups, implying that there was deception on the part of the participants demonstrating improper mask wear. Interestingly, there was no demonstrated difference in lying behavior among the other two groups, mask wearers and non-mask wearers (Tobol et al., 2020). This finding supports a correlation between lying behavior and improper mask usage. This may imply that people who improperly wear masks are more prone to lying, and could therefore be less socially conscientious. The resistance demonstrated by some of the population through lack of compliance to health protocols raises interesting questions regarding what variables might contribute to health compliance behavior. It is possible that influences from early life, such as the parenting style someone was raised under, could affect decisions to abide by or disobey COVID-19 mandates. Specifically, parenting style may influence how a person chooses to respond to COVID-19 health safety suggestions.

There are three primary parenting styles: Authoritarian, Authoritative, and Permissive (Baumrind, 1975). Each parenting style is described using a warmth spectrum and a discipline spectrum. Warmth refers to the affectionate love shown to the children, while discipline refers to how the parents modify unwanted behavior (Baumrind, 1975; Telep, 2014). High warmth would be described as caring about and paying attention to children’s feelings, offering physical or verbal comfort, expressing pride, admiration, and love verbally or physically, etc. Low warmth would be characterized by a lack of affectionate love; a parent who is emotionally distant (Baumrind, 1975).

Punishment refers to either physical or emotional direct harm imposed on someone to stop a behavior, and is often falsely synonymized with discipline. Conversely, discipline is the act of coaching someone to recognize why their behavior is problematic and how to better handle similar situations in the future (Telep, 2014). High discipline would be characterized by identifying a child’s problem behavior to them, explaining why the behavior is problematic, and working together to find a solution and to prevent the behavior in the future. On the other hand, low discipline would be characterized by allowing any behavior from the child, including problematic behavior, without repercussion (Baumrind, 1975; Telep, 2014).

Parenting styles may greatly affect the development of self-discipline, self-worth, social behavior, and a variety of other personality aspects (Baumrind, 1975, 2005). The type of parenting style experienced may also affect the development of conscience, or the awareness of morality regarding one’s behavior; a sense of right and wrong that urges one to act morally; a source of moral or ethical judgment or pronouncement (Wordnik, 2009).

Authoritarian parenting is characterized by strict rules and use of corporal punishment; low warmth and high punishment (Baumrind, 1975). The harsh, inconsistent discipline and lack of warmth common in Authoritarian up-bringing may increase the probability of children developing behavior problems. “In a vicious cycle, children’s poor performance may also heighten parents’ authoritarian behaviors” (Gao et al., 2014). Parenting styles which use domination, intrusion, and manipulation hinder psychological development and wellbeing in pre-adolescent children, and inconsistent discipline correlates with externalizing issues in preadolescent behavioral development (Baumrind, 1975). In other words, when parents do not consistently set boundaries or supervise, they are not modeling their expectations of the child (“do what I say, not what I do”). Further, inconsistent discipline leads to ignorance of or opposition to social expectations, how to treat others with respect, how to self-regulate, and hinders development of autonomy (Fuentes-Balderrama et al., 2020). Lastly, Authoritarian parenting styles have recently been found to be negatively correlated with executive control (Zhang et al., 2021).

Authoritative parenting is characterized by collaboration between parent and child; the parent acts as a guide rather than an unquestionable authority figure – high warmth and high discipline (Baumrind, 1975). The development of conscience early in childhood is correlated with mutual positive affect between mother and child, low maternal power assertion, and high maternal empathy (Kochanska and Aksan, 2006). Authoritative parenting techniques yield emotionally adjusted and socially aware children sooner in childhood than the other two parenting styles (Kochanska and Aksan, 2006). Additionally, Authoritative parenting has been the most effective at helping the development of personal responsibility (Bickley, 2022). Considering the COVID-19 pandemic guidelines are set for the safety of everyone, conscience may be a factor in whether people choose to obey or disobey those guidelines. In other words, someone who does not consider the feelings of others or how their actions may affect other people may be more likely to ignore the mandates due to their lack of empathy, social awareness, and moral compass.

In an Authoritative household, parents and their children communicate on an equal level (no extreme power dynamic), while the children are still being guided on how to behave in a socially acceptable way (Baumrind, 1975). Thus, people raised in Authoritative households are often able to handle distress without withdrawing or lashing out (Baumrind, 1975; Baumrind, 2005). Authoritative parenting is positively correlated with both children’s academic and social–emotional school readiness (confidence, self-efficacy, performance in language and cognitive development, communication skills, Chan and Koo (2011) general knowledge, emotional maturity, and social competence), but Authoritarian and Permissive parenting are negatively associated with children’s social–emotional readiness (Xia, 2020). Previous findings would suggest that participants reporting experience of Authoritative parenting would also report higher levels of Social Awareness than participants that do not.

Lastly, Permissive parenting is characterized by submission to a child’s whims and demonstration of a lack of guidance or discipline; high warmth, low discipline (Baumrind, 1975). Permissive parenting usually involves accepting and tolerating children’s impulsive behavior, thus not letting them properly develop self-regulation and social competence (Gao et al., 2014). This underdevelopment may lead to reckless behavior, inability to regulate emotions, and lack of concern for others (Baumrind, 1975; Baumrind, 2005). Thus, it is possible that participants reporting higher levels of both Social Awareness and Self-Regulation would be less likely to have experienced a Permissive parenting style.

While Authoritative parenting may yield more self-disciplined children that demonstrate social and emotional competence, Authoritarian and Permissive parenting may yield the opposite. This could have implications during the COVID-19 pandemic in that Social Awareness and self-discipline are both factors that may influence perception of a global pandemic and how people choose to behave during it. Parenting style experienced during childhood and adolescence may contribute to the level of adherence demonstrated to COVID-19 safety measures. Thus, previous research findings suggest that people with low social awareness, a lack of empathy, and low self-discipline would be more likely not to wear masks, not socially distance, etc. than people with properly developed social awareness and self-discipline (Tobol et al., 2020; Rahimi et al., 2021). Further, even if compliance was reported, it is possible that someone who experienced an Authoritative parenting style may adhere to health mandates due to feeling forced or feeling the need to obey authority, rather than out of empathy or consideration of others. Conversely, people that experienced Permissive parenting may also refuse to wear masks properly or at all, refuse to stay six feet away from other people, etc.

The current study employed a survey of questions related to both childhood experience and COVID-19 global pandemic behavior to observe the potential link between Parenting style and COVID-19 behavior. In addition, measures for both Social Awareness and Self-Regulation were also administered because of the strong connection between these variables and Parenting style that has been found in previous research (Baumrind, 1975; Kochanska and Aksan, 2006; Xia, 2020).

If parenting style experienced in childhood affected choice behavior, then participants identifying with an Authoritative parenting style should have scored higher on the COVID behavior measure when compared to participants that identified with Permissive parenting style, possibly due to Social Awareness and early development of conscience in childhood (Kochanska and Aksan, 2006). Thus, higher Social Awareness scores would also be expected from participants selecting Authoritative parenting than those that selected Permissive parenting.

Additionally, it was expected that participants that experienced an Authoritarian parenting style would report higher levels of COVID Behavior than participants identifying with Permissive parenting style, possibly due to lack of Self-Regulation/self-motivation (Baumrind, 1975, 2005) or less executive control (Zhang et al., 2021). If so, participants that identified with Authoritarian parenting would score lower on Self-Regulation than participants that selected Authoritative parenting.

Lastly, it was expected that participants that selected Permissive parenting style would score lower on COVID Behavior when compared to participants that identified with either Authoritarian or Authoritative parenting styles, possibly due to lack of accountability and responsibility (Baumrind, 1975, 2005). If so, participants that identified with Permissive parenting should have also demonstrated lower Social Awareness when compared to participants that selected Authoritarian parenting style.

## Methods

### Participants

Participants (*N* = 56) were Auburn University at Montgomery undergraduate students who were recruited from a subject pool using SONA Systems^1^ during the spring of 2022. Participation was voluntary and participants were given course credit for participating. All participants were over 18 years of age, with the average age of 20 and a range from 18 to 21. Participants identified as the following when asked for Gender and Race identification: (Male: 16; Female: 38; Other: 2), and (White: 20; Black/African American: 27; Other: 9).

### Procedure

The current study was administered through SONA Systems online (Sona Systems; https: sona-systems.com). The survey results were collected by Qualtrics.^2^ Before participating in the current study, students read and signed an informed consent form and were debriefed after completion of the study.

### Materials

This study employed items intended to measure Parenting Style experienced, Social Awareness, Self-Regulation, and COVID Behavior. To assess the parenting style participants reported experiencing in childhood, three vignettes (Authoritarian, Authoritative, and Permissive) were created in consideration of the traits of the different styles outlined by Baumrind (1975) and “Authoritative, Authoritarian, and Permissive Parenting Practices: Development of a New Measure” (Robinson et al., 1995).

Social Awareness was assessed by items were inspired by the SECQ (social emotional competence questionnaire) (Zhou and Ee, 2012). The scale used was a 4-point scale including the following choice options: 1 for “Never,” 2 for “Sometimes,” 3 for “Usually,” and 4 for “Always.” Example questions included: “I try to make other people feel comfortable around me,” and “I modify my behavior to fit different situations.” There were 10 items given to participants to measure Social Awareness. All 10 items were significantly correlated with the average score for Social Awareness items, so all were included in analyses including Social Awareness. Cronbach’s Alpha for the Social Awareness measure was *α* = 0.733.

Self-Regulation items used were modeled from the Self-Regulation Questionnaire (Carey et al., 2004). The scale used was a 4-point scale including the following choice options: 1 for “Never,” 2 for “Sometimes,” 3 for “Usually,” and 4 for “Always.” Example items included: “I tend to keep doing the same thing, even when it does not work,” and “I let others make decisions for me.” There were 20 items given to participants to measure Self-Regulation. Of the 20, 16 items were significantly correlated with the average score for Self-Regulation while four items were not. The four items that were not correlated with the average Self-Regulation score were omitted, thus 16 items were used in analyses including Self-Regulation. Cronbach’s Alpha for the Self-Regulation measure was *α* = 0.722.

To assess COVID Behavior, questions were created based off of the CDC COVID-19 safety suggestions (Centers for Disease Control and Prevention, 2020). The scale used was a 4-point scale including the following choice options: 1 for “Not True,” 2 for “Sometimes Untrue,” 3 for “Somewhat True,” and 4 for “Extremely True.” Sample items included: “Mask wearing in public is important,” and “I believe that social distancing is necessary.” There were nine items given to participants to measure COVID behavior. Of the nine, eight items were significantly correlated with the average score for COVID Behavior. The item that was not correlated with the average COVID Behavior score was omitted, thus eight items were used in analyses including COVID behavior. Cronbach’s Alpha for the COVID Behavior measure was *α* = 0.857.

## Results

### Descriptive statistics

The means, standard deviations, and correlation coefficients (Spearman’s Rho) are presented in Table 1 for all participants, in Table 2 for White participants, Table 3 for Other race participants, and Table 4 for African American/Black participants. For Gender, Male = 1 and Female = 2, and for Parenting Style, Authoritarian = 1 and Authoritative = 2.

**Table 1:** Descriptive statistics and correlation coefficients for all participants.

Condition	Mean	SD	2	3	4	5
Gender	1.704	0.461	0.069	0.118	0.765	0.02^*^
Parenting Style	1.778	0.42	–	0.781	0.497	0.022^*^
Social Awareness	2.978	0.424		–	0.481	0.958
Self-Regulation	1.89	0.318			–	0.773
COVID Behavior	2.975	0.728				-

^*^p < 0.05, ^**^p < 0.01.

**Table 2:** Descriptive statistics and correlation coefficients for White-Identifying participants.

Condition	Mean	SD	2	3	4	5
Gender	1.632	0.496	0.167	0.073	0.714	0.539
Parenting Style	1.842	0.375	–	0.666	0.249	0.033^*^
Social Awareness	3	0.325		–	0.87	0.471
Self-Regulation	1.99	0.274			–	0.405
COVID Behavior	2.54	0.788				–

^*^p < 0.05, ^**^p < 0.01.

**Table 3:** Descriptive statistics and correlation coefficients for other Race-Identifying participants.

Condition	Mean	SD	2	3	4	5
Gender	1.625	0.518	0.116	0.41	0.256	0.008^**^
Parenting Style	1.625	0.518	–	0.789	0.256	0.008^**^
Social Awareness	3.1	0.51		–	0.955	0.888
Self-Regulation	1.969	0.341			–	0.313
COVID Behavior	2.859	0.736				–

^*^p < 0.05, ^**^p < 0.01.

**Table 4:** Descriptive statistics and correlation coefficients for African American/Black-Identifying participants.

Condition	Mean	SD	2	3	4	5
Gender	1.778	0.424	0.723	0.096	0.608	0.019^*^
Parenting Style	1.778	0.424	–	0.406	0.669	0.864
Social Awareness	2.926	0.464		–	0.137	0.231
Self-Regulation	1.796	0.324			–	0.629
COVID Behavior	3.315	0.491				–

^*^p < 0.05, ^**^p < 0.01.

For all participants, there was a positive correlation between Gender and COVID Behavior (*r_s_* = 0.316, *p* = 0.02) and a negative correlation between Parenting and COVID Behavior (*r_s_* = −0.311, *p* = 0.022), indicating that participants that identified with Authoritarian parenting scored higher on the COVID Behavior measure. Looking more closely to discern if there were differences between participants in the different race categories, it was found that for White and Other Race-Identifying participants, Parenting Style was a negatively correlated with COVID Behavior (White-Identifying: *r_s_* = −0.490, *p* = 0.033; Other Race-Identifying: *r_s_* = −0.845, *p* = 0.008). Thus, participants identifying with the Authoritarian style and as White or Other race scored high on COVID Behavior. Gender was positively correlated with COVID Behavior for Other Race-Identifying participants (*r_s_* = 0.845, *p* = 0.008) and for African American/Black participants (*r_s_* = 0.449, *p* = 0.019), indicating that Female participants scored higher than Male participants on COVID Behavior for these two race categories.

### Parenting style

No participants selected that they identified with the Permissive Parenting vignette, so this reduced the number of parenting styles from three to two. 14 participants selecting the Authoritarian vignette while 42 participants chose the Authoritative vignette. Results from Mann–Whitney U Tests indicated that there was no effect of Parenting Style on Social Awareness (*U* = 266.5, *p* = 0.602) or effect of Parenting Style on Self-Regulation (*U* = 227, *p* = 0.204). However, there was an effect of Parenting Style on COVID Behavior (*U* = 152.5, *p* = 0.007), with participants that selected Authoritarian having a larger mean rank (38.61) than Authoritative participants (25.13).

### Gender

Two participants identified as “Other” when asked for Gender Identification. Those participants were omitted from the following analyses, which included participants identifying as “Male” or “Female.” Gender did not affect Social Awareness (*U* = 221.5, *p* = 0.117). or Self-Regulation (*U* = 288, *p* = 0.761). There was a significant difference between Male and Female participants on COVID Behavior (*U* = 183, *p* = 0.022), with female participants having a higher mean rank (30.68) than male participants (19.94).

### Race

Results from Kruskal-Wallis Tests indicated that there was no effect of Race Identification on Social Awareness (*H*(2) = 0.682, *p* = 0.711) or effect of Race Identification on Self-Regulation (*H*(2) = 5.51, *p* = 0.064). While the effect of Race was not significant on Self-Regulation (*p* = 0.064), it is possible that there might have been an effect in a larger sample size. Further exploration revealed no difference between White and Other Race participants (*U* = 88, *p* = 0.945) or between Other Race and African American participants (*U* = 72.5, *p* = 0.073), but a difference between White and African American participants (*U* = 176.5, *p* = 0.044).

Additionally, there was an effect of Race Identification on COVID Behavior (*H*(2) = 9.111, *p* = 0.011). Further examination using pairwise comparisons revealed that COVID Behavior did not differ between White participants and those that identified as Other Race (*p* = 0.842) or between participants that identified as Other Race and African American (*p* = 0.711). The significant effect of Race Identification on COVID Behavior was a result of the difference between White participants and African American participants (*p* = 0.008), with African American participants having a higher mean rank (34.85) than white participants (20.40).

## Discussion

The current study aimed to measure how parenting style experienced in childhood may impact reported behavior related to social awareness, self-regulation, and COVID compliance among college students. Because the Permissive parenting style employs little to no discipline and lacks the techniques needed to develop responsibility and empathy (Baumrind, 1975), it was hypothesized that lower scores on Social Awareness measures would correlate with a Permissive upbringing. However, none of the participants identified the Permissive parenting style as what they experienced during their upbringing, so the hypothesis could not be tested. The lack of Permissive parenting data may be a result of too much generalization in the Parenting Style identification portion of the survey. Perhaps measuring different traits of each Parenting Style instead of offering vignettes would provide more clarification on Permissive parenting, thus making it more identifiable. Focusing on Authoritative and Authoritarian parenting styles, it was hypothesized that higher scores on Social Awareness measures would correlate with an Authoritative upbringing as Authoritative parenting techniques often yield emotionally adjusted and socially aware children sooner in childhood than Permissive and Authoritarian parenting styles (Kochanska and Aksan, 2006). However, the present study found no evidence of a relationship between Parenting Style and Social Awareness.

Relatedly, participants with higher Self-Regulation scores were hypothesized to be more likely to have experienced an Authoritative upbringing because of the responsibility and autonomy usually fostered by this parenting style (Baumrind, 1975; Kochanska and Aksan, 2006). Conversely, it was hypothesized that lower Self-Regulation scores would more likely correspond with an Authoritarian upbringing due to the inconsistent discipline, domination, and manipulation (Baumrind, 1975) as well as a hinderance to the development of autonomy (Fuentes-Balderrama et al., 2020) experienced. Neither of these hypotheses were supported as no evidence of a relationship between Parenting Style and Self-Regulation was found.

Additionally, there was no relationship found between Social Awareness and COVID Behavior or between Self-Regulation and COVID-Behavior. However, a correlation was found between Parenting Style and COVID Behavior, indicating that participants who selected Authoritarian parenting scored higher on COVID Behavior relative to participants who selected Authoritative parenting. Further examination of this correlation revealed that the effect was present for White and Other race identifying participants but not for African American/Black participants. This finding supports that Authoritarian parenting may correlate with higher levels of compliant behavior, possibly due to less executive control (Zhang et al., 2021) or a reduced sense of autonomy (Fuentes-Balderrama et al., 2020) when compared to participants that reported experiencing Authoritative parenting. Another possibility is that perhaps participants that identified with an Authoritarian parenting experience felt more willing or able to question health guidance for COVID behavior because they have been able and/or potentially encouraged to question authority figures elsewhere, such as their home environment throughout their upbringing. In a survey study of adolescents in China, Bi et al. (2018) found that participants from an Authoritative household increasingly questioned parental authority figures as they matured. Putting this finding in the context of the current study, it is possible that participants that associated with an Authoritative parenting style scored lower on COVID behavior than their Authoritarian counterparts due to a propensity to question authority and recommended guidelines.

Focusing on the COVID Behavior measure revealed further differences between gender and race categories. When looking at all participants, females scored significantly higher on the COVID Behavior measure than males. There was a significant correlation between Gender and COVID Behavior for participants identifying as other race and African American/Black, but no correlation was present between Gender and COVID Behavior for White Participants. This suggests that female participants identifying as African American/Black or Other race reported more/higher COVID compliance than their male counterparts. Relatedly, a difference was found with regard to COVID compliance between race categories. Specifically, African American/Black participants scored significantly higher on COVID Behavior than participants in the other two race categories. Taken together, these findings support that African American/Black and Other race identifying participants reported more COVID compliance behavior, and further, that this effect could be driven largely by female participants within these race categories.

Lastly, while there was no significant difference found between race categories and Self-Regulation, the data suggested that this relationship was approaching significance and perhaps the sample size was too small to capture the effect if it was present. Further examination illuminated that White identifying participants scored significantly higher in Self-Regulation than African American/Black identifying participants. Thus, any relationship between race identification and Self-Regulation might be driven by the difference in self-report of Self-Regulation behavior between these two race categories.

It is important to acknowledge the limitations of the current study, which include its small sample size of college students as well as the collected data being representative of a specific time during the COVID-19 pandemic. However, the data reveal information about response to health guidance and what factors may influence compliance. Perhaps the most important findings were the ones that were not predicted in relation to the differences in reported behavior between different race and gender identifications. These two variables seemed to play large roles, especially with regard to COVID compliance. Future studies seeking to expand on the current findings should employ more in-depth questioning to examine why African American/Black participants and participants raised in Authoritarian households score higher on COVID Behavior, and if this higher level of compliance generalizes to other health guidance. Additionally, it would be beneficial to illuminate more on the higher scores from White identifying participants on Self-Regulation when compared to African American/Black participants. Specifically, if this effect is found consistently among participants of varying ages and what behaviors it might be related to.

Finally, the global pandemic has created a dynamic environment for collecting data, especially when the procedure focuses on the current status of the pandemic and the current health guidelines. The current study demonstrates findings from a set point in time during the COVID-19 pandemic by highlighting potential variables that may affect compliance to health guidelines. Having a better understanding of what factors contribute to behavioral choices during a health pandemic may help implementation of effective health guidance that more of the population will comply with.

## Data availability statement

The raw data supporting the conclusions of this article will be made available by the authors, without undue reservation.

## Ethics statement

The studies involving human participants were reviewed and approved by Auburn University at Montgomery Institutional Review Board. The patients/participants provided their written informed consent to participate in this study.

## Author contributions

AS contributed to developing this project, testing the measure used, data analysis, and writing this manuscript. JB supervised this project, contributed to its development, programmed the measure used, and contributed to data analysis and the writing of this manuscript. All authors contributed to the article and approved the submitted version.

## Funding

This study was partially supported by a grant from Auburn University at Montgomery. This was an internal grant that was awarded to support graduate student research.

## Conflict of interest

The authors declare that the research was conducted in the absence of any commercial or financial relationships that could be construed as a potential conflict of interest.

## Publisher’s note

All claims expressed in this article are solely those of the authors and do not necessarily represent those of their affiliated organizations, or those of the publisher, the editors and the reviewers. Any product that may be evaluated in this article, or claim that may be made by its manufacturer, is not guaranteed or endorsed by the publisher.

## Footnotes

^1^ www.sona.systems.com

^2^ www.qualtrics.com
